# Intergroup ‘Skype’ Quiz Sessions in Care Homes to Reduce Loneliness and Social Isolation in Older People

**DOI:** 10.3390/geriatrics5040090

**Published:** 2020-11-11

**Authors:** Sonam Zamir, Catherine Hennessy, Adrian Taylor, Ray Jones

**Affiliations:** 1 School of Nursing and Midwifery, University of Plymouth, Plymouth PL4 8AA, UK; 2 Faculty of Social Sciences, University of Stirling, Stirling FK9 4LA, UK; Catherine.hennessy@stir.ac.uk; 3 Peninsula Medical School, University of Plymouth, Plymouth PL4 8AA, UK; Adrian.Taylor@plymouth.ac.uk; 4 Centre for Health Technology, University of Plymouth, Plymouth PL4 8AA, UK; Ray.Jones@plymouth.ac.uk

**Keywords:** video calls, socialisation, action research, care-settings, Skype, intervention, loneliness, geriatrics, communication

## Abstract

Video calls using software such as Skype, Zoom and FaceTime can improve socialisation among older people and family, however it is unknown if video calls are able to improve socialisation among older people and their peers. Twenty-two residents across three British care homes engaged with each other using ‘Skype quiz’ sessions with the support of staff once a month over an eight-month trial. Video calls were accessed via a ‘Skype on Wheels’ intervention that comprised a wheeled device that could hold an iPad, or through Skype TV. Residents met other residents from the three care homes to build new friendships and participate in a thirty-minute quiz session facilitated by eight staff. Staff were collaborators who recruited older people, implemented the intervention and provided feedback that was analysed using thematic analysis. Residents enjoyed being able to see other residents’ faces and surroundings. Analysis of the field notes revealed five themes of: residents with dementia remember faces not technology, inter and intra connectedness, re-gaining sense of self and purpose, situational loneliness overcome and organisational issues create barriers to long-term implementation. Inter-care home connection through video calls to reduce feelings of loneliness in residents seems acceptable and a feasible, low cost model, especially during times of public crisis such as COVID-19.

## 1. Introduction

The COVID-19 pandemic has predominantly claimed the lives of those aged 60 and over, which is why strict isolation of older people has become a feature of many countries’ efforts to contain it [1]. While physical isolation may protect older people from the virus, it may increase distress from isolation. Internet access has benefits of keeping people connected to loved ones and forming new social ties beyond their home or care facility [2,3,4], but digital connectivity is not ubiquitous for older people who live in care [5]. Many older people in British care homes report feeling lonely and socially isolated [6] and this is likely to have increased due to the COVID-19 requirement to self-isolate. British care homes stopped any visitors for many months and some still limit the number of visitors to only a single designated person. This may have resulted in negative health outcomes such as depression, increased cognitive decline and even mortality [7,8,9,10,11].

Interventions employing digital technologies [12] such as Facebook or emails [13,14,15] and video-calls such as Skype, FaceTime and now Zoom [16,17,18,19,20] are one obvious way to tackle loneliness and social isolation in older adults. The need for such interventions reflects government policy ‘Digital by Default’ [21] that everyone, regardless of age or living arrangements, should be able to use the internet to access services, connect with healthcare professionals or socialise.

Although there is a wealth of research employing video-call technologies to help alleviate loneliness and isolation in older adults, many rely on familial social contacts [17,19,22], befrienders from a younger generation [23] or volunteers who may find it difficult to relate to older people who live in a care environment [24]. Unfortunately, not all family members can commit to video-call communication with their older relatives who live in care. Relatives may be older themselves and not possess the technology or skills to use video calls (digital deficiency). In other cases, relatives may live abroad and time differences and poor internet connectivity can be a problem [17].

Various initiatives have explored social contacts for older people with non-familial younger people via ‘inter-generational socialisation’ [23,25,26,27]. Although many studies have resulted in a positive impact for both age groups, including our own recent study on video-call sessions between care home residents and students (under review), video calls offer other possibilities.

Good quality, long-term relationships are often formed between those who have had similar experiences, likes, and dislikes, and therefore can better relate to one another. Despite common assumptions, some care home residents are capable of forming new friendships beyond the care home when given the opportunity [28]. Residents living in one care home might be expected to befriend one another and be content with these friendships. However, not all residents are able to form good quality friendships within their care home and so many feel isolated and lonely [17].

Importantly, discontinuing socialisation activities for those residents with dementia could potentially speed up cognitive decline and rapidly worsen the effects of dementia [19,29,30]. Care homes have encouraged staff to support residents to get ‘online’ to access social media tools such as Facebook and expand their social network virtually [5,13]. However, Porges’ social engagement and attachment theory [31] posits that face-to-face interactions are crucial for good quality social interactions and sustaining new and existing relationships. That is because body language and eye gaze influences important social cues that are clearly missed when interacting with individuals using text or social media platforms. Video calls can provide that much needed face-to-face social interaction.

Many care homes run quizzes for their residents as a form of entertainment and mental stimulation. We used the familiarity of quizzes for residents as a way of introducing video calls and engaging with other care homes. The current study aimed to explore whether inter-care home video calls were an acceptable and feasible intervention to reduce loneliness and social isolation for older people. The objectives were (1) to assess the feasibility and acceptability of using video calls to conduct an inter-care home quiz through Skype on Wheels (SoW) and/or Skype TV with older people living in care homes and (2) to determine whether non-familial social contact groups of the same age cohort are useful in increasing socialisation. The collaborative action research approach meant that care home staff and researchers worked together to try to achieve a successful outcome (i.e., to run the inter-care home quizzes successfully and to engage the residents). The outcomes of the study therefore were the simple yes/no views of the collaborators as to (i) whether it was feasible and acceptable, and (ii) if there were observed examples of what appeared to be increased socialization.

## 2. Materials and Methods

### 2.1. Design

The study employed a collaborative action research (CAR) design [32,33,34]. Within the CAR cycle there were five steps taken to achieve intervention implementation: (1) Recruitment of older people and relevant family. This was facilitated by staff in the care environment; (2) planning how best to implement the intervention. This required collaboration between the first author, staff, older people and their family; (3) implementation was the action of using video calls. (4) Reflection involved feedback and identification of the barriers to and benefits of using video calls; (5) re-evaluation allowed the researcher and staff to tackle the identified barriers. Observation was an on-going activity throughout the CAR steps and was integrated within the cycle.

### 2.2. Ethics

The study was approved by the University of Plymouth Ethics Committee in August 2016 (ID-15/16-582). All participants, care home staff and residents, gave written consent to participate and an information sheet was provided to all participants, and to resident’s next of kin. For those residents who were unable to provide written consent, care home staff obtained verbal consent prior to each video-call session and documented this in writing.

### 2.3. Care Home Sites

Three care homes (labelled C1, C4, C5) that were part of a larger CAR study for video-call use [17] continued their participation to this study which began in mid-2017. Our sample of care homes was therefore a convenience sample. Characteristics of each care site were documented (Table 1).

### 2.4. Participants

All residents had the opportunity to take part and 22 residents participated in video-call sessions. Our collaborators in the action research were eight care home staff who helped to facilitate the video calls and provided feedback. Characteristics of residents and facilitators were documented (Table 2).

### 2.5. Screening of Participants and Data Collection

Twenty residents had some previous experience of video call use two months prior to this study. These residents were part of the intergenerational study that connected them with students over a six-week trial (paper under review). An additional two residents were recruited to the study who did not want to take part in the intergenerational study, as they preferred to speak with social contacts their own age.

A feedback form was provided to care home staff to complete after each video-call session. Information such as who was called, length of call, number of residents engaged with and any technical problems was recorded. Our collaborating care home staff recorded if residents understood video calls, enjoyed their use, if they used the telephone handset and if they would like to continue using video calls. Care home staff provided either telephone feedback (one or two days after the video-call session) to the first author or face-to-face feedback. These were documented in writing and formed a set of field notes contributing towards qualitative feedback.

### 2.6. The Intervention

**Figure 1:**
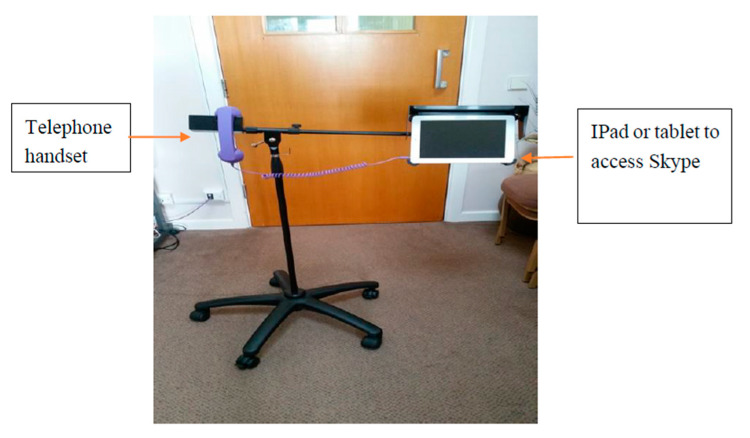
Skype on Wheels device.

**Figure 2:**
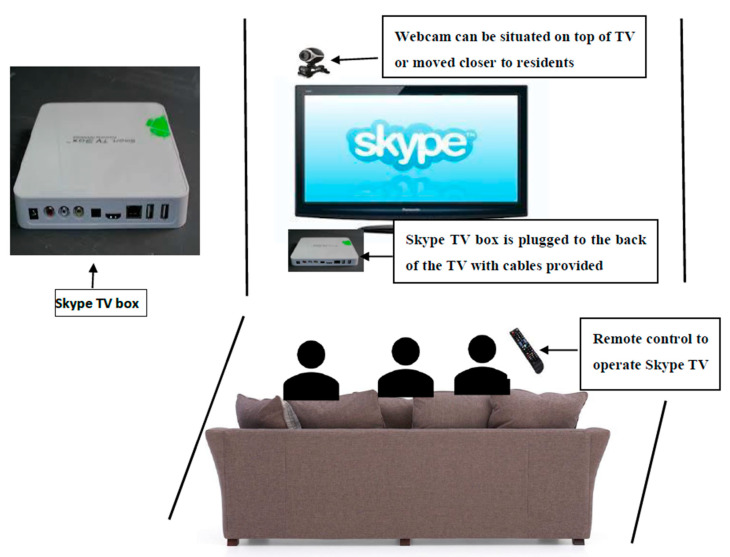
Typical set-up of Skype TV for sessions.

The Skype on Wheels (SoW) intervention was a simple portable device comprising an iPad to make video calls using free tele-services such as Skype, FaceTime or Zoom and a colourful handset (Figure 1). Skype TV was an alternative where a small box was connected to a TV in the communal area of the care home and a webcam was able to capture and transmit live video-calls onto the TV but was specific to the Skype software. A typical set-up using Skype TV is shown below (Figure 2).

### 2.7. Materials

In session one, a simple question and answer quiz was printed off by care staff in C1. This consisted of twenty questions that were relatively easy, for example, ‘what year did the second world war end in?’ or ‘is iron a metal?’. In subsequent sessions, each care home created their own version of the quiz which was approved by the researcher beforehand to ensure it was not too difficult, but also varied per session so the same questions were not repeated.

### 2.8. Procedure

Two months prior to the commencement of the study, an initial first test of this activity (session one) was conducted with C1 and C4. This was to test the feasibility of a ‘Skype quiz session’ (what care staff told residents the activity was called) to ensure it worked in practice, to identify whether SoW or Skype TV was better suited for the activity, and to identify any barriers that could quickly be tackled to ensure the smooth running of the study.

After this first test session, the participating care home agreed to a session once a month on a date of their choosing to begin with, and then once a fortnight towards the end of the study. Eight sessions were conducted in total as part of this study. Dates and times were agreed between the care homes and confirmed with the researcher through text message or email. A reminder call and/or text message was provided to each care home one week and one day before the session by the researcher. If a care home raised concerns such as the technology not working, the researcher visited the care home to test and help resolve technical issues before the next session was due. Sessions were held in the care home lounge of each participating care home before lunch and lasted for approximately one hour. On average there were six residents participating in a session.

The session typically began with 15–20 min of ‘meet and greet’ where residents could introduce themselves and make small talk to build friendships. After this, the ‘Skype quiz’ would begin with one care home staff member reading aloud the questions. This responsibility would alternate each session to ensure all care home sites had the equal chance to read their questions (for example, a staff member from C1 would read the quiz questions in session one, in session two a staff member from C4 would then read the quiz questions). Each question was read aloud three times giving one care home the chance to answer first correctly, if answered incorrectly the second care home had the chance to answer and so on. If answered correctly by the first care home, the next question would be answered by the second care home first and so on. A score was kept by staff or a nominated resident and the winning care home would be announced at the end of the quiz. After each session care staff participating provided short verbal feedback or through text message or telephone call to the researcher. Below is a descriptive outline of the sessions.

### 2.9. Session One: Test

The session lasted 40 min which consisted mainly of a quiz between the two care homes. The researcher was present at one of the care homes (C1) to help facilitate and document observations. Upon reflection of the first session, staff (*n* = 3) agreed that more time could be allocated to each session to include a ‘meet and greet’ between residents before moving onto the quiz. One care home (C1) used SoW and the other (C4) used Skype TV. It was decided that Skype TV was a more practically suited technology for the activity. This was because the larger screen of a TV was able to better capture and project a group of people that was needed for such an activity. The webcam part of Skype TV could be moved closer to an individual’s face when they were speaking and so was ideal for the ‘meet and greet’ part of the session that focused on individualised conversations between residents across the care homes.

### 2.10. Sessions Two–Five

Two care homes (C1 and C4) participated in the activity once every month, with the researcher alternating between them to facilitate and observe in session two, three and five. In session three, Skype TV was not working for C1 and so SoW was used as a back-up, however, this required more time and effort as it had to be continuously wheeled between participants.

### 2.11. Session Six

Three care homes (C1, C4 and C5) participated in the activity. The quiz was led by staff at C1, and an external staff member from Plymouth Museum was present with artefacts such as cleaning tools from the 1920s, and pictures of actors and iconic buildings from previous decades. These artefacts were brought up close to the Skype TV web cam in between the quiz questions and residents were asked what they could be.

### 2.12. Sessions Seven and Eight

Three care homes participated in the activity once every fortnight. The researcher was present at C4 for both sessions to help as staff were unable to use Skype TV due to technical problems. At session eight the researcher announced to all participating care homes that it would be the last session in the study, however they were welcome to continue independently.

### 2.13. Data Collection

An ethnographic approach consisting of observations, informal unstructured feedback, memo writing and semi-structured interviews was taken [35,36]. The researcher documented all observations in note form. All conversations between collaborators and participants were anonymised and documented into memos after each visit in a retrospective format. A semi-structure interview guide for both residents and care staff was developed by the researcher in the first instance. The interview guide for residents was then presented to one care home manager and one activities coordinator who felt it was necessary to shorten the interview from 30 min to 20 min as to not exhaust residents (especially those with dementia), unless residents decided to speak for longer. After a test interview with one female resident, the questions were altered to become more directed towards video calls and the activities to avoid residents going off topic.

### 2.14. Data Analysis

Thematic analysis was used to analyse the field notes and interview transcripts by the first researcher [37]. Saturation sampling was used, in which observations and interviews stopped when no new dominant issues or themes were found emerging from the data. The data was analysed in a precise, concise and exhaustive manner to establish trustworthiness of the qualitative results. The main researcher and one supervisor analysed and coded the transcripts (including rotation). These were then reviewed by the full supervisory team. The naming and checking of the categories, final themes and appropriate quotes were done by all of the researchers and the supervisory team. The software package NVivo version 12 was used to organise and manage the data.

## 3. Results

### 3.1. Overall Findings

Documented observations and consistent feedback from care staff revealed the importance of ‘technology type’, ‘checking equipment’, ‘competitive activities’ and ‘peer interactions’ to ensure that the activity would be successful over a long period.

Staff feedback revealed that Skype TV was a preferred method for this activity as SoW was not always able to capture and project the full size of the group from one care home to another. Care staff felt it was too ‘time consuming’ to continuously wheel around SoW between residents during the activity. Nonetheless, SoW worked well during the ‘meet and greet’ part of the activity as this was more individualised. Care staff also reported that reminders a week in advance would prompt them to check the equipment and report any technical issues rather than leaving it too late. This also enabled care staff to feel ‘more responsible’ towards the intervention equipment by ensuring it was kept somewhere safe, that it had full power (charging battery of iPad or changing battery in Skype TV remote) and that user logins were easily retrievable.

The competitive aspect of the quiz became prominent after session three as observations and care staff feedback revealed that residents became more eager to video call in the lead up to the next session as winning became ‘our homes pride’. Similarly, each care home had noticeable ‘top star’ residents who were able to answer questions correctly during the quiz. This in turn helped residents from the other home to remember their names, faces and even their backgrounds. For example, one ‘top star’ resident revealed that he used to work as a teacher; this was previously unknown even to residents from within his own care home. This resident who was a teacher became well known to the others throughout the remainder of the sessions.

As the sessions progressed, many of the same residents would continue to participate, but also fellow residents (from within the care home) would observe and decide to participate in the next session if they had not yet done so. This improved peer interactions within each care home to help build friendships and recruit residents to future sessions. Peer interactions across care homes improved vastly from session three to session eight as residents began to remember each other and engage in more meaningful small talk, for example, asking about each other’s families, their fashion and the way their care homes were different or similar.

#### 3.1.1. Themes

Follow-up interviews with participating care staff and residents revealed five key themes with twelve corresponding codes (Table 3) which are discussed below.

##### A. Residents with Dementia Remember Faces Not Technology

Aa. Unrecognisable Technology

Participants with moderate to advanced dementia (who were able to communicate through interview) did not remember using video calls for communication. During the interview when shown the intervention equipment to help prompt them, they did not recognise SoW or Skype TV, with two insisting they have never used them.

Ab. Remember Conversations

Although residents with dementia did not recognise the technology, they were able to remember having conversations with people ‘outside’ of their care home and answering questions in a ‘game’. However, not all residents remembered the faces of the individuals they spoke with, and two became confused between conversations with school pupils (from a previous study) and the quiz activity. For example, two residents began to talk about a conversation they were having with a school pupil almost six months before but insisting it was a recent conversation as part of the quiz activity. When told that those conversations were not recent (with school pupils), two residents became distressed and conversations about socialisation ceased, and the interview focus shifted to the type of video-call technology (if they liked SoW, Skype TV and technology in general). The other residents who were asked about socialisation were able to remember the competitiveness of the quiz and some faces of residents in other homes, but again, not using the technology.

“*I don’t think so, not used this before. Yes it was good because* [resident] *answered everything and won, good to have a team member like that … yes it was done through … oh I don’t know … not on this or that … like normally*”.

(Resident with dementia)

Ac. Expressing Positive Emotions

Residents expressed feelings of happiness when they remembered having conversations with individuals outside of the care home. They were able to recall what the content of the conversation, the gender of the social contact and even the clothing they had worn during the conversation. One resident remembered the activity coordinator from a participating care home during the activity, describing her ‘purple clothes’, glasses and ‘lovely smile’.

“*Oh yes it was a lot of fun something new and I was excited for it … she was … you know* [resident] *lovely and hair like mine sometimes she wore the lilly that was interesting*”.

(Resident with dementia)

##### B. Inter and Intra Connectedness

Ba. Increased Socialisation within the Home

Socialisation appeared to be two-fold, with residents increasing their conversations with fellow residents (inter) and forming new social contacts across care homes (intra) during the ‘meet and greet’ aspect of the activity. Interconnectedness improved the quality of their social ties with fellow residents as they learnt more about each other’s backgrounds and interests, which were unknown before the start of the activity. Residents also spoke fondly about their ‘teammates’ during interviews and explained how they recently learnt they have things in common. C4 care home residents appeared to be more closely connected to their fellow residents before the start of the activity compared to the other homes, however residents still expressed feeling more connected with each other during the activity.

“*I couldn’t believe* [resident]! *He was on fire that time answering everything we didn’t have the need for anything thinking …* [resident] *was very knowledgeable usually very quiet to himself never shared but I guess no one asked him before this. Probably other residents who I don’t know much about also*”.

(Resident)

Bb. Increased Socialisation Across Homes

Some residents were able to remember the names of residents across care homes, but only those who had participated in all sessions. Three residents mentioned how surprisingly similar their care homes were in terms of the furniture, lounge set-up and even weekly activities they tended to engage with. These comparisons were a popular conversation among residents, improving the intra-connectedness across the homes. Overall, residents felt comfortable interacting with other care home residents as they were able to relate to them and did not feel they had to filter the conversation as they had done with the school pupils (intergenerational study under review). Finally, one resident explained she had told her family about the activity and how she was able to meet similar people, thus increasing her social networks.

“*It’s always nice to see a new face … I mean yes the kids were all talkative and interesting but we all felt we had to be mindful of what were said … you know. They are much younger so we spoke about newer topics and they asked a lot of questions … maybe for homework … with what we did (the quiz) it’s good to see others like me*”.

(Resident)

“*Oh yes I spoke to my daughter and told her about this and she was just, very pleased oh yes very pleased she can’t wait to see how it all works*”.

(Resident)

##### C. Sense of Self and Purpose

Ca. Opportunity to Share Stories

Residents reported that video calls allowed them to not only see new faces but gave them the opportunity to share their own life stories with people of a similar age. Rather than engaging in conversations that were mostly about sharing knowledge and giving advice to a younger generation, they were able to talk about life events that happened with people who had also experienced it.

“*The children were lovely they showed me their library and spoke about their projects but it was different I would say. With this* (quiz)*, we spoke about our lives and even when I used to live up country because* [resident] *also did. I got to share with them … someone new who is happy to hear!*”.

(Resident)

Cb. Remember Their Past Selves

Using the technology helped prompt memories of when residents had first engaged with technology in their past. One female resident disclosed that she used to work within the air force where she first came across computers for communication and made comparisons between old technology and Skype TV and SoW. Another female resident explained that her husband had worked for British Telecom (BT) and how they had always been so interested in technology, however when entering in care without her husband she had become disinterested in her old interests, such as technologies, until now.

“*It was different very basic then, but it had a key purpose if we didn’t use it, such huge problems for the work, we had no choice. When I first came across … it was amazing … felt like such an expert! This box was able to communicate from up there … but now yes it is similar but the technology has changed. Had we been able to see a faces then … well I doubt we could have it was too old*”.

(Resident)

Residents had begun speaking about their past in relation to technology, but also other stories among the peers within their care home which increased their weekly socialisation. Similarly, as the sessions progressed, they felt comfortable sharing their past across the care homes and also remembered information concerning other residents’ pasts.

Cc. Insecurities

Two residents still expressed some insecurities about their image which caused a deterrent to want to possibly continue their participation in future video-call sessions. Both residents had been using video calls for at least six months now on a regular basis (in the previous inter-generational study and the Skype quiz) yet worried that others may not like their image, or the way they look. One resident said at times he did not like video calling as he did not feel comfortable with ‘just anyone’ seeing him. Instead he suggested that when he felt this way, he could simply just move away from the screen.

“*But then they can see your face and sometimes you just don’t want anyone to notice your big nose, or unwarily hair or … you know. You can hide in here so I don’t know. Not every will like you*”.

(Resident)

“*I didn’t like it too much. But actually everyone liked each other and if you don’t like someone you can just move and not participate. Yes see what it is all about … I did enjoy it*”.

(Resident with dementia)

##### D. Overcoming Situational Loneliness

Da. Overcome Boredom

Most residents across the three care homes explained that video calls for socialisation helped them to ‘pass the time’, and gave them ‘something to do’. This reason was indicative to why some of the older residents (80 years and over) were keen to participate as ‘what else is there to do at this age?’

“*Good to see them face-to-face, something to do … I know it’s not good to speak to people you don’t know … but … shes a talker. Maybe it’s good to use on certain occasions when with friends something to see. I don’t have a house or wife and the years go by now*”.

(Resident)

Db. Relate to Others

The group activity within the care home allowed residents to have a common experience with their peers, thus being able to relate to one another more closely and increasing their connectedness. One resident explained that before she had not really participated in any of the care home activities and felt slightly like an ‘outsider’ keeping to her room. The quiz activity brought her closer to her fellow residents where now she felt included and comfortable, but she also enjoyed being able to see new faces across the care homes.

“*I’ve always kept myself to myself you come here people already have their own groups you just sit watch a bit of telly* (TV) *and pass the time without really even knowing anyone. We have something to talk about even other things now and then you see the other people … you think maybe I could go there*”.

(Resident)

One female resident in C1 was very surprised to be able to speak to a resident ‘across the bridge in Cornwall’ who was originally from the same city as her before she moved to Devon. Other residents were surprised that there were so many people who had similar professions as them such as a teacher, a nurse or working for the military.

##### E. Organisational Issues Cause Barriers to Long-Term Implementation

Ea. Staff Availability and Support

Care staff felt that this time round (cycle two), they were now familiar with the technology and enjoyed it because the activities were a result of the staff recommendations after cycle one. Therefore, they felt more involved in each step of the process and responsible towards committing to each video-call session. Nonetheless, this did not help overcome the organisational issues within their care home, such as lack of staff to support the activities, changes in staff roles meaning less time for activities and most importantly the lack of time they have to ensure video-call activities continue regularly.

“*This was an amazing innovative initiative which all care homes should now get on board with. It worked a lot better this time round compared to last year I think because the staff now … they got the hang of it. It does take a bit of time but it’s worthwhile. Only problem I can see it … not with the technology we can use it now … not with the residents even the families are getting on board … they like the activity. But it’s just staff to support this. Families need us to focus on the care, the physical care and even then we are low on staff. Maybe if we had some more support even external support I can see this continuing*”.

(Care home manager)

Eb. Desire to Implement Long-Term

Because staff were now more involved in each video-call activity compared to cycle one, they were able to see the positive effects of video-call socialisation on their residents, both with and without dementia. Being directly involved in the quiz activity, rather than simply supporting residents by holding or moving a device, was particularly beneficial in seeing the impact of such an activity. Care staff themselves enjoyed taking part and highlighted that the competitive nature of the activity (quiz) made them want to continue it each month. Similarly, they liked being able to see and speak to care staff from across each care home where they could also share stories and ‘get to know each other’. Staff felt that video calls through this activity could actually help care homes to ‘link up’ and become more connected with each other to provide a more ‘close knit’ unit.

“*Yes we loved the quiz it was really competitive and actually it felt like the entire home was involved in each session … because it’s a matter of the care homes pride! No but it’s all good fun and games and at first I was thinking gosh I will never get the idea of this it won’t last but with the help we can actually see how much the residents, the whole home loves it*”.

(Activities coordinator)

“*I would say it is really good because even for us staff we get to connect up with our sister homes. Yeah it was competitive and we wanted to win but actually it’s good to know what other homes are doing and get some tips and share stories. We feel more like a connected community of homes*”

(Activities coordinator)

Furthermore, care staff explained that the video-call activities had been shared with residents’ families, which in turn prompted and encouraged family members to video call their relatives. One family member in C1 decided to attend a quiz session to ‘witness firsthand’ and reported positive feedback, but also the need to ‘continue on with this’.

Care staff directly experiencing the benefits of the activity increased their desire to implement the use of video calls in the long-term. One activity coordinator suggested that video calls should be implemented through a series of activities such as first to ‘dress-up’ the technology, then to try out with school pupils (intergenerational study under review) for a short time, then to begin regular quiz sessions with other care homes and finally to use video calls to connect with distant relatives, or even other organisations such as a church on Sundays. Another activity coordinator explained that their care home would now want to ‘link up’ through the quizzes with their eight sister homes in the region and set it up as a competition with rewards for the winning care homes.

“*We’ve already had a discussion about this and were going to try and link up with about eight maybe even nine of our sister homes across this region for the quizzes. I think it would be good for them to so it in the stages that we did it because it worked*”.

(Activities coordinator)

## 4. Discussion

The idea of connecting to multiple care homes through video calls for socialisation can appear to be complex in its set up and implementation, especially when involving people with dementia. Although this study used a small number of cases, it evidenced that a ‘link up’ of multiple care homes through video calls using Skype or possibly FaceTime and now Zoom is a feasible and acceptable activity for socialisation for older people, helping them to stay connected even during times of public crisis that require self-isolating. However, Skype TV was the preferred technology for this activity as it was able to better capture a larger group. 

A key objective was addressed in this study; it found that other care home residents are a useful non-familial social contact to video call and thus increased residents’ social networks. Simultaneously, the activity was able to retain older people to the study, allowing a prolonged use of video calls. Furthermore, this study is the first to connect two or more care homes through video calls for socialisation over a long period. All of the participants including care staff felt video calls for socialisation were a compelling component of the quiz activity and indicated they were interested in continuing with this on a regular basis, highlighting the longevity of the intervention. The newer Zoom software [20] for video calls is catered to larger social gatherings and has been the choice for many individuals who have been working from home or self-isolating due to COVID-19. This study suggests that video calls, specifically Skype, work well for residents with and without dementia, yet further research needs to be done explore the Zoom software in a care setting. Facebook TV portal would be very suitable technology for such links.

The study supports other research that has employed e-health technology, similarly finding that video calls can form a network of peer support in older people and shape positive new relationships within the same age cohort [38]. However, we have not found other studies that are direct parallels of our work as we first included people with dementia; second, connected to more than two care sites virtually in real time; and third, embedded a quiz activity that has never been tested prior to this research. There is a need for additional investigations to replicate this research to draw comparisons, and to inform conclusions on the usefulness of an inter-care home socialisation activity that can be adopted by others.

Group members who engage in regular face-to-face communication have been known to still establish uniformity in beliefs and actions as an important source of social validation [39,40]. Group socialisation activities have been considered useful as groups give more information than a single individual, and so a group can tap into a wider variety of backgrounds and interests to keep conversations interesting. Groups stimulate creativity and can problem solve far better than a single individual [39,40]. Thus, the quiz component became equally enjoyable for residents as they were able to work together to answer questions. Individuals tend to remember a group discussion or activity better, as group learning is known to foster improved learning and comprehension. Individuals in small groups tend to learn more and retain information longer when the same materials and exercises are presented to them in other formats [41,42]. This may be why a quiz provided through video calls in a group setting was so well accepted by older people with and without dementia.

An important finding to present in this study was that residents with dementia did not remember using the video-call technology, however remembered communicating with new people. Some even remembered key features of the social contact such as their gender, hair or clothing. It has now become well-known that those with dementia can recall how an event made them feel even if they are no longer able to remember the faces or names. Studies even suggest that those with more advanced dementia who become non-verbal should be able use non-verbal communication as an alternative as many are able to process distinct emotions such as happy and sad faces [43]. This recommendation fits with the key theme of ‘residents remember faces not technology’ found in this study. This produces an even further compelling need for researchers and care staff alike to include people with dementia in new innovative interventions that can improve well-being.

Some older people living in care seemed to regain a sense of self and purpose again. This may be tied in with the theme of situational loneliness that was present in the data where many of the individuals expressed their need for something engaging to do to pass their time. In other studies, older people have recommended that in order to reduce loneliness, various forms of interaction and activities in which communication is predominant are preferred [44]. Other initiatives in the UK to reduce loneliness in older people include a network of 70 ‘friendship clubs’ [45]. Through transport and venue provision older people are able to meet locally and engage in activities supported by facilitators such as card games, information giving sessions and informal conversations. However, this initiative relies on funding for transport and venue to continue, highlighting a possible drawback for those who are unable to leave their home. Yet, this initiative does provide support for this study, clearly demonstrating that loneliness can be tackled through group face-to-face socialisation with an embedded activity [45]. The current study did the same but virtually, meaning a cost savings on travel and venue.

Although the activity demonstrated improved socialisation, the intervention may have been ‘disguised’ as a socialisation activity as the quiz aspect of the study was the ‘selling point’ for both residents and care staff. Participating in a quiz was something that was familiar to participants as it was something they had all previously engaged with. Therefore, residents may have had an increased liking for this activity due to the quiz component rather than the socialisation component. This ambiguity needs to be further explored to distinguish if answering questions in the quiz or speaking to new faces was a contributing factor towards wanting to continue participation. Simultaneously, there is ambiguity on whether residents wanted to continue their participation due to the new friendships they made across the care homes through video calls, or whether they enjoyed the company of their fellow residents in their group (intra versus inter socialisation). The study did not measure for the effects of inter socialisation (within the care home) against intra socialisation (across care homes) which could be a significant contributing factor to consider for future trials in deciding the effectiveness of the video-call intervention. Specifically, it is possible that inter socialisation coupled with the quiz, and not the act of video-calling others, produced benefits. Other researchers have noted this issue and attempted to tackle it, for example, differentiating the effects of group socialisation and reminiscence activities [46].

As compared to our previously published research [17] and our intergenerational study (under review), this study included the largest set of participants. One possibility may be because not all residents felt comfortable speaking to a younger generation (intergenerational study under review) and actually there are certain aspects of socialisation that need to be taken into consideration that contribute to successful socialisation. For example, forming numerous direct, high quality ties to people who appear more valuable and beneficial to an older person takes precedence for successful socialisations [47]. The idea of forming an ‘egocentric’ network appears decidedly important for older people as higher density networks where individuals know each other well constitute to a close-knit social tie. This is where individuals can triangulate information, interests and resources [47]. The inter and intra socialisation was prominent in this study as individuals were eager to form new social ties with people who they had something in common with such as interests and even backgrounds. Establishing an ‘egocentric’ network may prove to be more difficult with a younger generation as compared to peers of the same age.

The need for care staff or a facilitator for this activity, as with the other video-call activities in this research, is still crucial. This can appear to be a large drawback in successfully reducing loneliness for older people where the intervention relies heavily on staff availability and their self-efficacy in technology use. Although a number of studies are being conducted in complex care environments through better collaboration with the care staff, there are still organisational issues that are difficult to tackle to effectively implement innovative interventions that address important health outcomes. The first stage is to improve the negative attitudes that can arise from care staff in adopting additional care duties to tackle outcomes such as loneliness. Working with care staff and ensuring they were closely involved in each step of the collaborative process improved their attitudes towards video calls, their self-efficacy and desire to implement. Care staff also felt they were part of the activity as they had an important role to read out the quiz questions. Therefore, they were not simply adjusting the technology for older people during a session, or a bystander.

## 5. Conclusions

Video calls connecting one care home with another for quiz sessions provide a stimulating and recognisable activity that can help residents with and without dementia engage in socialisation, thus offering the opportunity to increase and sustain their social networks.

## Figures and Tables

**Table 1:** Characteristics of care sites.

	C1	C4	C5
No. of care staff at site	45	40	15
Care staff participating	2	3	3
Education level of staff (highest level)	College	College and undergraduate degree level	College
Average number residents	30	40	17
Specialist care type	Dementia	Dementia	Frailty
Video-call equipment available in care home	iPad Samsung Galaxy tablet Skype TV SoW device	iPad Skype TV SoW device	iPad SoW device

**Table 2:** Characteristics of older participants and facilitators.

Participant	Age	Gender	Previous Experience of Video-Calls	Dementia or Signs of Cognitive Decline	Physical Disabilities
Residents N = 22	65+	Male = 5 Female = 17	Yes = 20 No = 2	N = 7	Hearing impaired * = 12 Visually impaired ** = 9 Non-verbal *** = 3 Frailty **** = 6
Facilitators N = 8	22–50	Male = 2 Female = 6	Yes = 3 No = 5	n/a	n/a

Note: * Need for or wears hearing aid ** Need for or wears glasses *** Unable to articulate verbally and/or uses sign language **** Poor mobility such as in a wheel chair and/or unable to independently walk/get up/hold heavy objects without assistance.

**Table 3:** Themes and codes.

Theme	Code
A. Residents with dementia remember faces not technology	Aa. Unrecognisable technology Ab. Remember conversations Ac. Express positive emotions
B. Inter and intra connectedness	Ba. Socialisation within the home Bb. Socialisation across homes
C. Re-gaining sense of self and purpose	Ca. Opportunity to share knowledge Cb. Remember their past selves Cc. Insecurities
D. Situational loneliness overcome	Da. Overcome boredom Db. Relate to others
E. Organisational issues cause barrier to long-term implementation	Ea. Staff availability and support Eb. Desire to implement long-term

## References

[1] Loneliness Endangers the Body as Well: How to Stay Connected with Elderly Loved Ones. https://www.theguardian.com/society/2020/mar/31/elderly-people-coronavirus-stay-connected?CMP=Share_iOSApp_Other.

[2] Cotten S.R., Anderson W.A., McCullough B.M., Perlman D., Jordan-Marsh M. (2013). Impact of Internet Use on Loneliness and Contact with Others among Older Adults: Cross-Sectional Analysis. J. Med. Internet Res..

[3] Chang B.L. (2004). Internet intervention for community elders: Process and feasibility. West. J. Nurs. Res..

[4] Morahan-Martin J., Schumacher P. (2003). Loneliness and social uses of the Internet. Comput. Hum. Behav..

[5] McCardle L. Carehome.co.uk Reveals over 80 per Cent of Care Home Residents Are Missing out on ‘crucial’ internet. https://www.carehome.co.uk/news/article.cfm/id/1560819/plea-to-get-residents-online-as-research-reveals-thousands-are-missing-out-on-crucial-internet-access.

[6] Loneliness and Social Isolation in the United Kingdom. https://www.campaigntoendloneliness.org/loneliness-research/.

[7] Adams K.B., Sanders S., Auth E.A. (2004). Loneliness and depression in independent living retirement communities: Risk and resilience factors. Aging Ment. Health.

[8] Aylaz R., Aktürk Ü., Erci B., Öztürk H., Aslan H. (2012). Relationship between depression and loneliness in elderly and examination of influential factors. Arch. Gerontol. Geriatr..

[9] Laloux J.L. (2014). Loneliness and cognitive decline. Soins Gerontol..

[10] Holt-Lunstad J., Smith T.B., Baker M., Harris T., Stephenson D. (2015). Loneliness and Social Isolation as Risk Factors for Mortality. Perspect. Psychol. Sci..

[11] Luo Y., Hawkley L.C., Waite L.J., Cacioppo J.T. (2012). Loneliness, health, and mortality in old age: A national longitudinal study. Soc. Sci. Med..

[12] Chen Y.-R.R., Schulz P.J. (2016). The Effect of Information Communication Technology Interventions on Reducing Social Isolation in the Elderly: A Systematic Review. J. Med. Internet Res..

[13] Jung E.H., Sundar S.S. (2016). Senior citizens on Facebook: How do they interact and why?. Comput. Hum. Behav..

[14] Osman Z., Poulson D., Nicolle C. (2005). Introducing computers and the Internet to older users: Findings from the Care OnLine project. Univers. Access Inf. Soc..

[15] Chipps J., Jarvis M.A., Ramlall S. (2017). The effectiveness of e-Interventions on reducing social isolation in older persons: A systematic review of systematic reviews. J. Telemed. Telecare.

[16] Easy Video Calling for Seniors with KOMP. https://www.noisolation.com/uk/komp/easy-video-calling-for-seniors-with-komp/.

[17] Zamir S., Hennessy C.H., Taylor A.H., Jones R.B. (2018). Video-calls to reduce loneliness and social isolation within care environments for older people: An implementation study using collaborative action research. BMC Geriatr..

[18] Skype for the Elderly Smart Video Calling. https://smart-life-solutions.co.uk/skype-for-the-elderly.

[19] Boman I.-L., Lundberg S., Starkhammar S., Nygård L. (2014). Exploring the usability of a videophone mock-up for persons with dementia and their significant others. BMC Geriatr..

[20] Zoomphone. https://zoom.us/.

[21] Government Digital Strategy-Report. https://www2.ed.gov/digitalstrategy/index.html.

[22] Hensel B.K., Parkeroliver D., Demiris G. (2007). Videophone Communication Between Residents and Family: A Case Study. J. Am. Med. Dir. Assoc..

[23] Wenzel M.P.S., Rensen S.S. (2000). Changes in attitudes among children and elderly adults in intergenerational group work. Educ. Gerontol..

[24] Dodge H.H., Zhu J., Mattek N.C., Bowman M., Ybarra O., Wild K.V., Loewenstein D.A., Kaye J.A. (2015). Web-enabled conversational interactions as a method to improve cognitive functions: Results of a 6-week randomized controlled trial. Alzheimer’s Dement. Transl. Res. Clin. Interv..

[25] Cohen G.D. (2000). Two new intergenerational interventions for Alzheimer’s disease patients and families. Am. J. Alzheimer’s Dis..

[26] Canedo-García A., García-Sánchez J.-N., Pacheco-Sanz D.-I. (2017). A Systematic Review of the Effectiveness of Intergenerational Programs. Front. Psychol..

[27] de Jong Gierveld J., Dykstra P.A., Schenk N. (2012). Living arrangements, intergenerational support types and older adult loneliness in Eastern and Western Europe. Demogr. Res..

[28] Sabat S.R., Lee J.M. (2012). Relatedness among people diagnosed with dementia: Social cognition and the possibility of friendship. Dementia.

[29] Fratiglioni L., Paillard-Borg S., Winblad B. (2004). An active and socially integrated lifestyle in late life might protect against dementia. Lancet Neurol..

[30] Moyle W., Jones C., Cooke M.L., O’Dwyer S., Sung B., Drummond S. (2014). Connecting the person with dementia and family: A feasibility study of a telepresence robot. BMC Geriatr..

[31] Porges S.W. (2003). Social engagement and attachment. Ann. N. Y. Acad. Sci..

[32] Taylor A.H., Everson-Hock E.S., Ussher M. (2010). Integrating the promotion of physical activity within a smoking cessation programme: Findings from collaborative action research in UK Stop Smoking Services. BMC Health Serv. Res..

[33] Kemmis S. (1993). Action research and social movement. Educ. Policy Anal. Arch..

[34] Kemmis S., McTaggart R., Nixon R. (2013). The Action Research Planner: Doing Critical Participatory Action Research.

[35] Fetterman D.M. (2009). Ethnography: Step-By-Step.

[36] Hammersley M., Atkinson P. (2007). Ethnography: Principles in Practice.

[37] Clarke V., Braun V., Hayfield N. (2015). Thematic Analysis. Qual. Psychol. Pract. Guide Res. Methods.

[38] Ezumi H., Ochiai N., Oda M., Saito S., Ago M., Fukuma N., Takenami S. (2003). Peer support via video-telephony among frail elderly people living at home. J. Telemed. Telecare.

[39] Keller M., Festinger L., Schachter S., Back K. (1950). Social Pressures in Informal Groups, a Study of Human Factors in Housing.

[40] Kruglanski A.W., Shah J.Y., Pierro A., Mannetti L. (2002). When similarity breeds content: Need for closure and the allure of homogeneous and self-resembling groups. J. Personal. Soc. Psychol..

[41] Barkley E.F., Cross K.P., Major C.H. (2014). Collaborative Learning Techniques: A Handbook for College Faculty.

[42] Davis R.L. (1997). Group Work is NOT Busy Work: Maximizing Success of Group Work in the L2 Classroom. Foreign Lang. Ann..

[43] Luzzi S., Piccirilli M., Provinciali L. (2007). Perception of Emotions on Happy/Sad Chimeric Faces in Alzheimer Disease: Relationship with Cognitive Functions. Alzheimer Dis. Assoc. Disord..

[44] Azeredo Z.D.A.S., Afonso M.A.N. (2016). Loneliness from the perspective of the elderly. Rev. Bras. Geriatr. Gerontol..

[45] Gardiner C., Geldenhuys G., Gott M. (2018). Interventions to reduce social isolation and loneliness among older people: An integrative review. Health Soc. Care Community.

[46] Haslam C., Haslam S.A., Jetten J., Bevins A., Ravenscroft S., Tonks J., Haslam S.A. (2010). The social treatment: The benefits of group interventions in residential care settings. Psychol. Aging.

[47] Cornwell B., Laumann E.O., Schumm L.P. (2008). The Social Connectedness of Older Adults: A National Profile. Am. Sociol. Rev..

